# Reproduction strategies of the silver birch (*Betula pendula *Roth) at post-industrial sites

**DOI:** 10.1038/s41598-021-91383-0

**Published:** 2021-06-07

**Authors:** Izabella Franiel, Agnieszka Kompała-Bąba

**Affiliations:** 1 Faculty of Natural Sciences, Institute of Biology, Biotechnology and Environmental Protection, University of Silesia in Katowice, 9 Bankowa Street, 40-007 Katowice, Poland; 2 Faculty of Natural Sciences, Institute of Biology, Biotechnology and Environmental Protection, University of Silesia in Katowice, 28 Jagiellońska Street, 40-032 Katowice, Poland

**Keywords:** Ecology, Plant sciences, Ecology, Environmental sciences

## Abstract

The study aimed to evaluate the parameters of reproductive traits, specimens’ fertility and reproductive efficiency observed in *Betula pendula* populations growing at different types of sites (zinc-lead heaps, coal mine heaps and unpolluted site). The leaf biomass and the biometric characteristics of inflorescences and fructifications were identified. Moreover, the biometric parameters of *B. pendula* seedlings were evaluated for examined sites. Seed-originated trees mostly of age 40 were randomly selected and from each tree, a branches from 1.70 m height and orientation N–S, W–E to the cardinal points of the stem were chosen. In the laboratory, selected soil parameters, the viability of pollen and the seeding value of seeds were analysed. According to the multidimensional statistical analysis the populations of *B. pendula* growing on post-industrial wastelands represent different morphotypes with lower values of almost all the reproductive traits, compared to the unpolluted birch population. Such traits as the male:female catkin number ratio and the non-embryo seed number were positively correlated with the heavy metal content at the zinc-lead heaps; at the same time these traits were negatively correlated with soil fertility. The fully developed seed number and the mature female catkin number were strongly correlated with the available potassium and phosphorus soil content but also with the leaf number on the generative shoots. The specimens of birch growing in these three habitats did not develop a universal reproductive strategy. Some differences in fecundity, the condition of seeds and the patterns of seed germination were found. The resulting seedling survival is determined by the plasticity of biometric traits, sheltered places for germination, etc. Seedlings that originated from heaps (local gene resources) are more suitable for use in the reclamation of large amounts of waste.

## Introduction

Mechanisms of development and changes of organisms, population, and species have been known in outline ^1–4^, but supplementary detailed studies are still needed to combine morphological traits, soil properties and ecological variation particularly in post-industrial habitats that can be treated as a kind of novel ecosystems ^5^. It is known that different types of environmental stresses (acid or basic pH, low organic matter and nutrient content, low water availability, high concentrations of heavy metals Cd, Cr, Fe, Ni, Pb, Zn), elevated CO_2_ and CO_3_ that plants suffer from can influence the suite of life history traits of species that successfully colonise post-industrial sites and form stable vegetation cover ^6–14^. They should be considered as a kind of compromise between allocation of energy to growth, development and reproduction ^15–17^. Variation in plant traits leads to the fitness of individuals under diverse environmental conditions. Structuring of resource availability, and the strategies used to garner resources, are critical predictors of interspecific competition and coexistence within communities ^18^. Several models were constructed in order to determine the relationships between environmental factors and vegetative vs. generative reproduction in herbaceous and woody plants ^7^. In herbaceous species, mainly root growth was reduced, while aboveground biomass did not change (the decrease in leaf size was compensated by an increase in leaf number). In contrast, in case of woody plants (mainly trees and shrubs) no changes were recorded in allometry whereas their growth and reproduction was reduced to a greater extent than growth of herbaceous plants ^16^. As a result of stress and decrease in environmental capacity, generative reproduction investment should decrease along with an increase in pollution. Moreover, poor quality seeds can then be produced, which will result in poor germination and low seedling survival. Many authors have even reported sexual dimorphisms in the life history traits of trees, such as reduced vegetative growth ^15,19,20^ and less frequent flowering in females than in males. However, not all species show the relative reproductive cost between the sexes^21^. The efficiency of propagation before the reproductive stage is affected mainly by individual fertility, as well as by progeny viability ^22,23^. The survival of individuals and their reproductive success are a consequence of the (morphological, physiological) traits modified by natural selection, as well as by miscellaneous adaptation strategies ^8^. Since trees are long-lived, differences between genotypes are particularly important in coping with environmental stresses. Some populations respond to deteriorating life conditions with increased reproductive capacity. However, after exceeding a certain level of stress, reproduction does not take place at all ^24,25^. Most studies on the responses of trees to their environment relies on short-term experiments with young plants under controlled conditions. However, how species adapt to environmental stresses, can change with ontogeny and can be different between laboratory (controlled) and natural growing conditions ^26^.

In this study, we have addressed these issue by examine relationships between generative traits and environmental factors in the silver birch (*Betula pendula* Roth) a habitat-forming species appearing transiently in the initial course of succession ^27–31^. To our knowledge, most research on birch trees concerning their adaptation to disturbed habitats and areas under harmful industrial emissions have dealt with morphological and ecophysiological traits and, to a lesser degree, reproductive potential ^32,33^. Birches growing on heavy metal (e.g., nickel, copper) polluted sites are smaller, have different life forms and growth patterns (slow growth), as well as different leaf size and distribution in comparison to those from unpolluted sites ^34,35^.

The aim of the study was to assess whether:

– the potential for sexual reproduction differs between birch populations growing on seminatural (unpolluted) and post-industrial sites,

– stress caused by heavy metals and lack of nutrient enhances generative reproduction traits,

– seedling emergence and quality are limited by seed availability.

We expected that stress caused by the heavy metal contamination or lack of water which birch trees suffer from in post-industrial sites will enhance generative reproduction.

## Materials and methods

### Study sites

The research was conducted in 2011–2013 in the Silesian Upland (in its Katowice Upland mesoregion), southern Poland. Precipitation ranges from 700 to 800 mm. The average annual temperature is ca. 8.2 °C (average January temperature is − 1.5 °C, and July 17.6 °C) ^36^. As a result of the mining of mineral resources (hard coal, zinc, lead and iron ores, sand, gravel, and dolomites) that began in the early Middle Ages and further processing of lead and zinc ores by smelting, a variety of waste and spoil has been stored ^37^. For further research we chose three types of habitats: slag heaps in Katowice Wełnowiec; Siemianowice Chemiczna Street; Świętochłowice Lipiny ca. 30 years after the dumping had finished and where pioneer vegetation was observed (Zn-Pb D H1-H3); coal mine spoil areas—the KWK “Murcki” heap, the KWK “Boże Dary” heap, the KWK “Kostuchna” site (Coal mine D K4-K6) and the transition zone between mixed forest and meadows in the Katowice Forest Park, which is a large wooded area in the southern part of the Katowice area (Control L L7–L9) (Table 1). The plant material was formally identified by corresponding author Izabella Franiel and confirmed by Prof. Adam Rostański, Head of the Laboratory of Botanical Documentation and Scientific Herbarium of the University of Silesia in Katowice, where the collected material have been deposited.

**Table 1:** Locality and characteristics of the waste material from Zn-Pb dumps, coal mine dumps and control sites.

Type of habitat	Geographical coordinates	Locality	Characteristics of the waste material and control sites
Zn–Pb D H1	N5016′57′′; E1901′09′′	Katowice-Wełnowiec, non-ferrous Metallurgical Plant “Silesia”	The waste material consists mainly from slag coming from blast muffle, cinder from distillation and roasting furnaces with mixed and fine granulometric composition
Zn–Pb D H2	N5017′02′′; E1902′26′′	Siemianowice, Chemiczna Street	
Zn–Pb D H3	N5018′17′′; E1853′29′′	Świętochłowice, Lipiny	
Coal mine D K4	N5011′23′′; E1902′08′′	KWK “Murcki” dump	The substratum of the heaps were carboniferous rocks (clay, shales, sandstones)
Coal mine D K5	N5011′06′′; E1900′39′′	KWK “Boże Dary” dump	
Coal mine D K6	N5010′48′′; E1859′18′′	KWK “Kostuchna” dump	
Control L7	N5014′03′′; E1901′19′′	Katowice Forest Park (unpolluted site)	Transitional zone between mixed forest and abandoned meadows; fluvisols and brown soils
Control L8	N5014′42′′; E1900′13′′	Katowice Forest Park (unpolluted site)	
Control L9	N5013′59′′; E1901′28′′	Katowice Forest Park (unpolluted site)	

### Biometric traits of *B. pendula* generative organs

At each of the 9 study sites, 10 trees were randomly selected for detailed studies. The seeded trees we studied were 17 m tall, mostly of age 40 and had fully developed female and male catkins (the age class from 20 to 50)*.* We chose classification of the age of trees according to the Forest Data Bank,where the age of trees is classified into I–V (a and b) age groups (0–20, 20–40 years of age etc.), and for mature (seeded) trees this class is IIa–IIIa (20 to 50 years of age) https://www.bdl.lasy.gov.pl/portal/. The tree age was assessed by ^38^. Since the birch does not necessarily from annual rings every year in polluted areas, we decided to use a non-invasive method to determine the age of trees since we can quickly assess the age by measuring the tree height on the basis of ready-made tables or growth curve diagrams [see ^29,39,40^]. All measurements were taken with the Suunto PM-5/1520 Altimeter (Finland). From each tree, a twig was randomly selected, 1.4–2.1 cm in diameter, with generative long and short shoots (ca 100 twigs from each study site). The number of leaves (LN) on each selected birch twig was counted at the end of June. The biometric characteristics of birch inflorescences and fructifications were measured with an electronic calliper and were noted at the beginning and at the end of the growing season. They included the number of male catkins (MCN), the number of female catkins (FCN), the male:female catkin ratio (MCN:FCN), male catkin length (MCL) [cm], female catkin length (FCL) [cm]; the number of fructifications on short shoots: the number (MtFCN) and the length (MtFCL) [cm] of mature female catkins, and nuts (seeds): the number of non-embryo seeds (N-ESN) and the number of fully developed seeds (FDSN). During birch pollen production, pistillate and staminate inflorescences were counted for each selected twig but the length of staminate inflorescences was determined at the end of winter.

### Soil analysis

We collected 10 soil samples from 0 to 20 cm horizons at each site (90 in total) for analysis of the physicochemical parameters. They were pooled, mixed, air-dried, and then ground and sieved through 2 mm mesh. The soil pH was measured electrometrically in water (pH in H_2_O) and 1 M KCl suspension (1:2.5 w:v). The organic carbon content was estimated using the Tiurin method. Total N content was assessed by the Kjeldahl method using Automatic Kjeldahl Digestion Units and UDK 129 Kjeldahl DKL Distillation Units (VELP Scientifica, Italy). The total content of heavy metals (Cd, Pb, Zn) and available Mg, K, Ca cations in the substrate were determined by flame atomic absorption spectrometry (Varian Spectra AA 330) after hot digestion of 0.25 mg of soil in a mixture of 65% HNO_3_ and 35% H_2_O_2_ (8:2) with the ETHOS ONE microwave system (Milestone, S.r.l., Italy). Exchangeable forms of metals in soil were determined by shaking 5 g of soil in 50 ml of 0.01 M CaCl_2_ for 2 h. Soluble (available) phosphorus in soil was determined by the Egner-Riehm method. Exchangeable basic cations were extracted with 1 M ammonium acetate pH 7.0 and their concentration in the extracts was determined by absorption (Mg^2^ +) or emission (Ca^2 +^, K ^+^, Na ^+^) spectrometry ^41^.

### Variability of pollen and seed germination

In the laboratory the variability of pollen and the seeding value of seeds were analysed. Experiments were carried out immediately after the collection of plant material in April and September every year. Staminate inflorescences (in total 80 from each site in every vegetative season) were collected in the field during the growing seasons (2011–2013) in order to examine pollen viability. The pollen was brushed off from anthers onto microscope slides with nutrient agar (prepared from a mixture of sucrose and “Difco” agar in a ratio 1:2.5 in distilled water) and was placed in a glass chamber with over 90% humidity at the temperature of 21 °C. After 1, 2, 3 and 24 h the growth of a pollen tube was observed. Germinated pollen grains (pollen germination capacity in %) were counted in 10 replications, 50 grains each, in the visual field under a Motic BA210 light microscope ^11^. In August of each growing season, ten fructifications were collected from every selected twig. The length of each fructification and the total number of nuts were determined in the laboratory, dividing the nuts into two categories: empty (without embryos) and fully developed ones, using a binocular microscope (Motic BA210). The sowing of seeds was performed immediately after the collection of fructifications. For every research plot, 12 Petri dishes (9 cm in diameter) with two layers of filter paper were prepared, onto which 25 birch seeds were sown and placed in a thermostat at 23 °C and with a 12 h light/12 h dark photoperiod (light flux: ca. 120 micro mol/m^2^s) and 50% relative humidity. Germinated seeds were counted every day for two weeks. A seed was considered to be germinated if the seed radicle was at least half the length of the nut. Based on the data obtained, the germination power of seeds (seed germination percentage) and the mean germination time (MGT) were used ^42–44^. The seed germination percentage was calculated as: G = G_i_/G_t_ × 100%, where G_i_ represents the number of germinated seeds 14 days later, and G_t_ represents the total number of seeds of each replicate. MGT is defined as a measure of the rate and time span of germination and it is equal to Sum (n x t)/Sum n, where t is the time from the beginning of the germination test in days, and n is the number of newly germinated seeds at time t. MGT (unit: day) is just the index of germination speed. The low value of this index means fast germination and high seed vigour ^45,46^.

### Biometric traits of birch seedlings

In order to evaluate the biometric traits of birch seedlings, 2 m × 70 m research plots were established at each of the study sites (Zn–Pb D H1–H3; Coal mine D K4–K6; Control L L7–L9). To facilitate the observations, they were divided into smaller 1 m^2^ plots. Field studies were carried out in each September of the two vegetative seasons. Using an electronic calliper, the biometric traits of *B. pendula* seedlings (in total 50 plants over 5 cm high in each September) were determined. Observations and biometric measurements were applied to the followings plant traits: the height of a plant – the distance from the ground level up to the tip of the longest shoot (shoot length); the number of branches (shoot number); the number of leaves on the leader shoot (number of seedling leaves).

### Statistical analyses

Ordination analyses were used to evaluate the influence of environmental factors on the chosen biometric traits of *B. pendula* from the polluted (Zn–Pb D, Coal mine D) and the unpolluted sites (Control L). Principal component analysis (PCA) was used in order to find patterns in the *B. pendula* biometric traits. The gradient length was 0.4, indicating the linear structure of the data, so the next step was the redundancy analysis (RDA). RDA was followed by the forward stepwise selection of environmental variables and the unrestricted Monte Carlo test with 499 permutations. All ordination analyses were performed using the CANOCO 5.0 software ^47^.

To examine the effects of the site (Zn–Pb D, Coal mine D and Control L) on plant biometric traits, one-way ANOVA was used. The data were log(x + 1) or arcsine transformed in order to meet the assumptions of ANOVA for normality and homogeneity of variance. If ANOVA indicated significant differences, Tukey’s HSD test was used to determine whether differences between sites were significant (p < 0.05).

To compare the pollen germination capacity and the quality of birch seeds (MGT and G indices) between the research areas, the Kruskal–Wallis test and the non-parametric multi-comparison test were performed. We used non-parametric tests because data did not meet the conditions of the normal distribution. All analyses were performed with Statistica version 13 ^48^, assuming the significance level α  = 0.05.

The biometric traits of *B. pendula* seedlings (shoot length, number of shoots, number of leaves) were analysed statistically with the Generalized Mixed Models (Restricted maximum likelihood method) in R ver. 3.3.0 ^49^(R Development Core Team 2008) with the research site (Zn–Pb D, Control L) as a fixed variable (fixed effect) and the vegetative seasons (2012–2013) treated as a random variable (random effect).

### Ethics approval and consent to participate

All methods in the present research were performed in accordance with the relevant guidelines and regulations specified in BMC journals’ Editorial policies.

The present research did not involve human or animal participants.

### Research involving plants

The research and field studies on plants presented in this paper comply with relevant institutional, national and international guidelines and legislation. The research did not involve rare or endangered species of fauna or flora, or species at risk of extinction.

The *Betula pendula* is a common species in Poland; it is not a protected species under national conservation laws and no permissions or licenses are required for the collection of the plant specimens or seeds.

## Results

### Relationships between biometric traits of *B. pendula* populations from polluted and unpolluted sites and environmental factors

**Figure 1:**
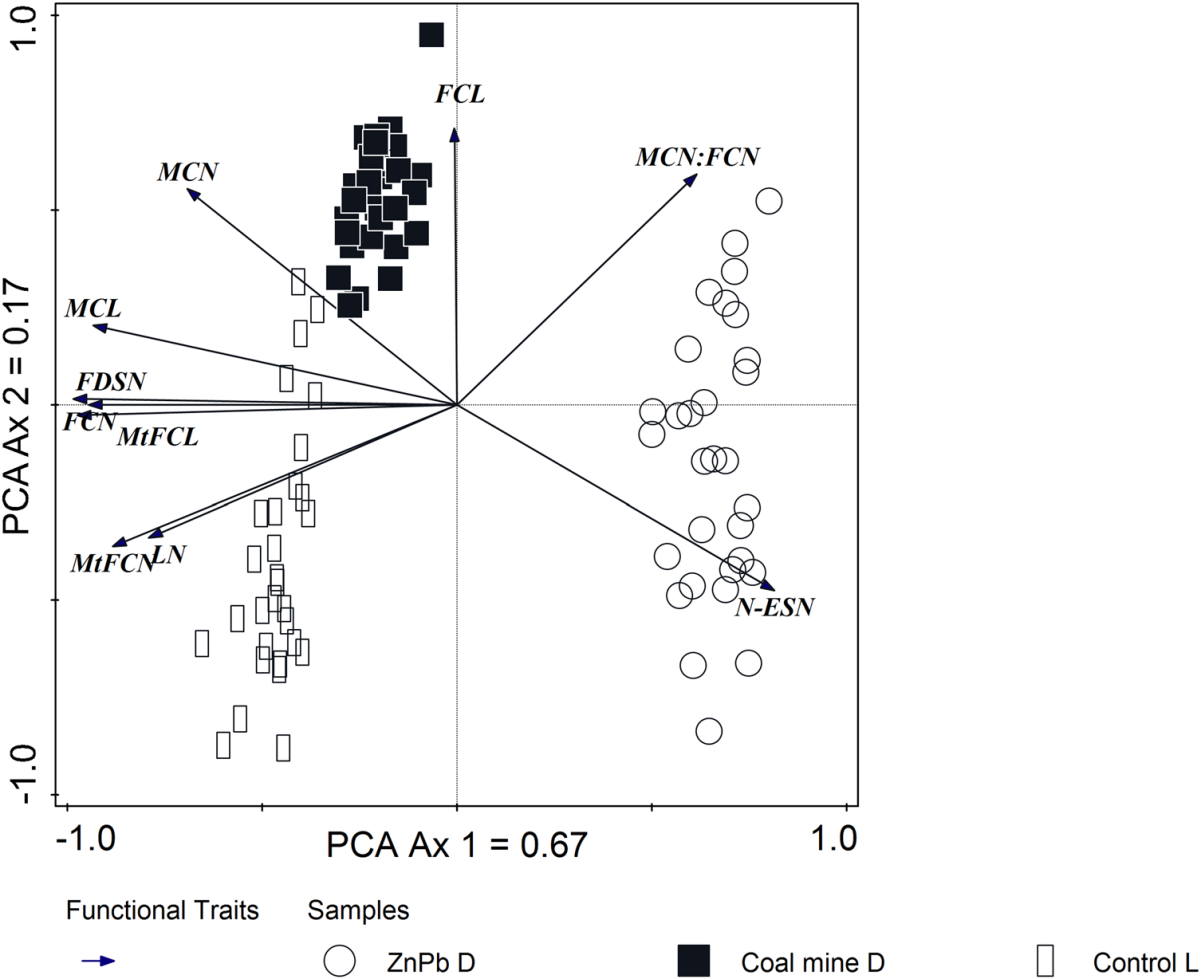
PCA analysis of the differentiation of the biometric traits of *Betula pendula* on Zn-Pb D, Coal mine D and unpolluted Control L sites. The traits abbreviations are given in Table 2a.

The Zn–Pb D silver birch populations, with the lowest values of all of the measured traits, differed significantly from other populations: the Coal mine D and unpolluted sites (Control L) (Table 2a). The latter population had the highest number of female catkins as well as male catkin length and the number and length of mature female catkins. Moreover, in this population the highest number of leaves on the branch was recorded in comparison to both post-industrial sites (Fig. 1). The highest male catkin number, as well as female catkin length, was recorded for Coal mine D population in comparison to other sites. The male:female catkin ratio was the highest in the case of Zn–Pb D, whereas the lowest value of the ratio was counted for the Control L population. The highest number of non-embryo seeds, as well as the lowest number of fully developed seeds, was recorded in the silver birch population taken from the Zn–Pb D (Table 2a).

**Table 2:** Descriptive statistics (mean and standard deviation) and the results of Tukey’s HSD test: (a) the biometric traits of *B. pendula* populations; (b) the soil properties from the Zn–Pb D, Coal mine D, Control L sites (significance level α = 0.05; a different lower-case letter means significant differences).

(a)				
Biometric traits	Abbreviation	Zn–Pb D	Coal mine D	Control L
Number of leaves on the branch	LN	247.56 ± 34.71a	313.26 ± 38b	412.26 ± 78.97c
Number of male catkin	MCN	29.00 ± 8.41a	57.70 ± 6.06b	44.26 ± 7.64c
Number of female catkin	FCN	18.63 ± 5.91a	39.56 ± 5.04b	50.03 ± 6.28c
Male:Female catkin ratio	MCN:FCN	1.64 ± 0.55a	1.46 ± 020a	0.85 ± 0.13b
Male catkin length (cm)	MCL	1.99 ± 0.19a	2.93 ± 0.11b	3.06 ± 0.14c
Female catkin length (cm)	FCL	2.30 ± 0.11a	2.53 ± 0.08b	2.26 ± 0.40a
Number of mature female catkin	MtFCN	253.63 ± 26.92a	327.66 ± 17.50b	474.28 ± 41.51c
Number of non-embrio seeds	N-ESN	188.36 ± 19.72a	70.31 ± 7.05b	98.45 ± 8.02c
Number of full developed seeds	FDSN	65.27 ± 12a	257.35 ± 15.81b	375.83 ± 42.66c
Mature female catkin length (cm)	MtFCL	1.96 ± 0.16a	3.51 ± 0.11b	4.36 ± 0.13c

(b)				
Physicochemical soil parameters	Unit	Zn–Pb D	Coal mine D	Control L
pH (in KCl)		6.47 ± 0.17a	6.11 ± 0.12b	6.76 ± 0.01c
C organic carbon	g/kg	227.04 ± 16.86a	426.87 ± 12.70b	182.50 ± 3.16c
N total	g/kg	3.41 ± 0.24a	6.14 ± 0.37b	2.80 ± 0.12c
C to N ratio		67.45 ± 0.85a	69.54 ± 1.57b	67.06 ± 3.34a
Available K_2_O	mg/kg	54.93 ± 10.46a	145.95 ± 22.75b	240.15 ± 23.35c
Available P_2_O_5_	mg/kg	7.10 ± 1.25a	6.41 ± 0.74a	43.55 ± 1.69b
Available MgO	mg/kg	24.07 ± 8.21a	10.41 ± 1.48b	26.10 ± 0.48a
Exchangeable Na^+^	mmol/kg	0.65 ± 0.03a	0.64 ± 0.03a	1.81 ± 0.53b
Exchangeable K^+^	mmol/kg	2.05 ± 0.03a	4.22 ± 0.52b	7.06 ± 2.22c
Exchangeable Ca^2+^	mmol/kg	32.69 ± 0.11a	47.75 ± 22.71b	71.01 ± 0.07c
Exchangeable Mg^2+^	mmol/kg	0.25 ± 0.03a	0.69 ± 0.17b	24.02 ± 11.36c
Cd	mg/kg	141.93 ± 3.91a	17.65 ± 3.37b	0.02 ± 0.01c
Pb	mg/kg	14,566.35 ± 61.29a	198.46 ± 1.43b	284.09 ± 12.34c
Zn	mg/kg	40,410.78 ± 151.21a	265.85 ± 5.08b	46.05 ± 2.94c

**Figure 2:**
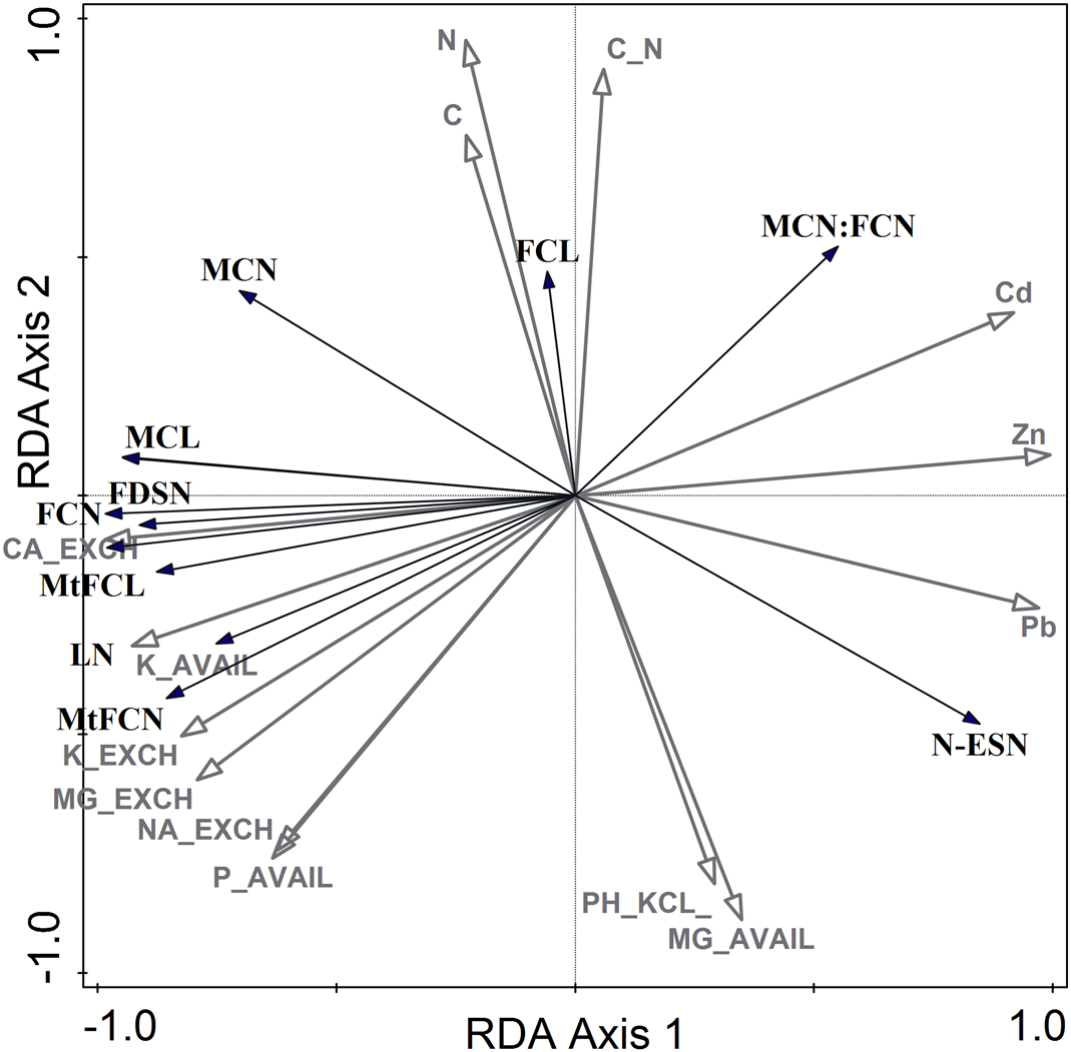
RDA biplot presenting the biometric traits along the vectors that represent the gradient of the environmental variables. All abbreviations are given in Table 2.

Redundancy analysis (RDA) (Fig. 2, Table 2b) revealed significant relationships between environmental variables and the birch biometrical traits. All the canonical axes explain 89.26% of the total variation in the measured traits. The highest percentage of variation is explained by the first axis (81.67%). The Monte Carlo test revealed that the variability of the birch fitness traits was significantly related to the first RDA axis (F = 334, p = 0.002), and all the canonical axes were significant (F = 45.5, p = 0.002). The forward stepwise selection of variables showed that the total zinc content (explain 64.70% of the total variation, p = 0.002) and available phosphorus (explain 11.70% of the total variation, p = 0.002) significantly influenced the silver birch traits.

The ordination of the measured traits and sampling points along the vectors representing the gradients of the environmental variables is presented in the biplot (Fig. 2). The first RDA axis is strongly positively correlated with Cd (r = 0.919), Pb (r = 0.970) and Zn (r = 0.995) in soil and negatively with available potassium (r = − 0.928), available phosphorus (r = − 0.635) as well as exchangeable forms of Na, K, Ca, Mg. The second RDA axis is strongly positively correlated with C (r = 0.755), N (r = 0.955) and C:N (r = 0.894) and strongly negatively correlated with available magnesium (r = − 0.862) and pH (r = − 0.705).

The silver birch traits male:female catkin number ratio and non-embryo seed number were positively correlated with the heavy metal content at the Zn–Pb D site, and at the same time negatively correlated with the soil fertility. The opposite trend is visible in the Control L population. The other traits were positively correlated with the macroelements in the soil. The fully developed seed number and the mature female catkin number were strongly correlated with the available potassium and phosphorus soil content but also with the leaf number on the generative shoots.

Negative correlations were detected between heavy metal content (Zn, Pb, Cd) in the substratum and such traits as number of leaves on the generative shoots, male catkin length, male and female catkin number, mature female catkin number and length, or the number of fully developed seeds. By contrast, positive correlations were found between the abovementioned traits and the exchangeable forms of Na, K, Ca, Mg or the available forms of potassium K_2_O and phosphorus P_2_O_5_.

Positive correlations were found between the male:female catkin number ratio, the number of non-embryo seeds and the elevated level of heavy metal content in the substratum. Furthermore, positive correlations were found between the C and N total content and the male catkin number, female catkin length and the male:female catkin number ratio, whereas there were negative correlations between the numbers of non-embryo seeds (Fig. 2).

### Pollen variability

Pollen germination capacity differed significantly between the examined sites. A significantly lower germination capacity was recorded in the case of pollen from the Zn-Pb D (median = 2.26), in comparison to pollen from the Coal mine D (median = 69.66). The highest pollen germination capacity was recorded for the Control L site (median = 98.03) (Table 3).

**Table 3:** Results of the non-parametric multi-comparison test of *B. pendula* viability indices (pollen capacity and mean germination time (MGT), germination percentage (G) of seeds from Zn–Pb D, Coal mine D and Control L sites (the significance level α = 0.05; a different letter means significant differences).

Viability indices	Zn–Pb D			Coal mine D			Control L		
	Min	Max	Median	Min	Max	Median	Min	Max	Median
Pollen capacity (%)	0.34	28.98	2.26a	62.18	81.15	69.66b	92.64	99.81	98.03c
MGT (days)	1.04	1.24	1.14c	0.81	0.91	0.82a	0.90	0.95	0.93b
G (%)	10.40	21.60	20.00a	25.20	36.80	31.60b	40.00	47.40	42.20c

### Seed germination

**Figure 3:**
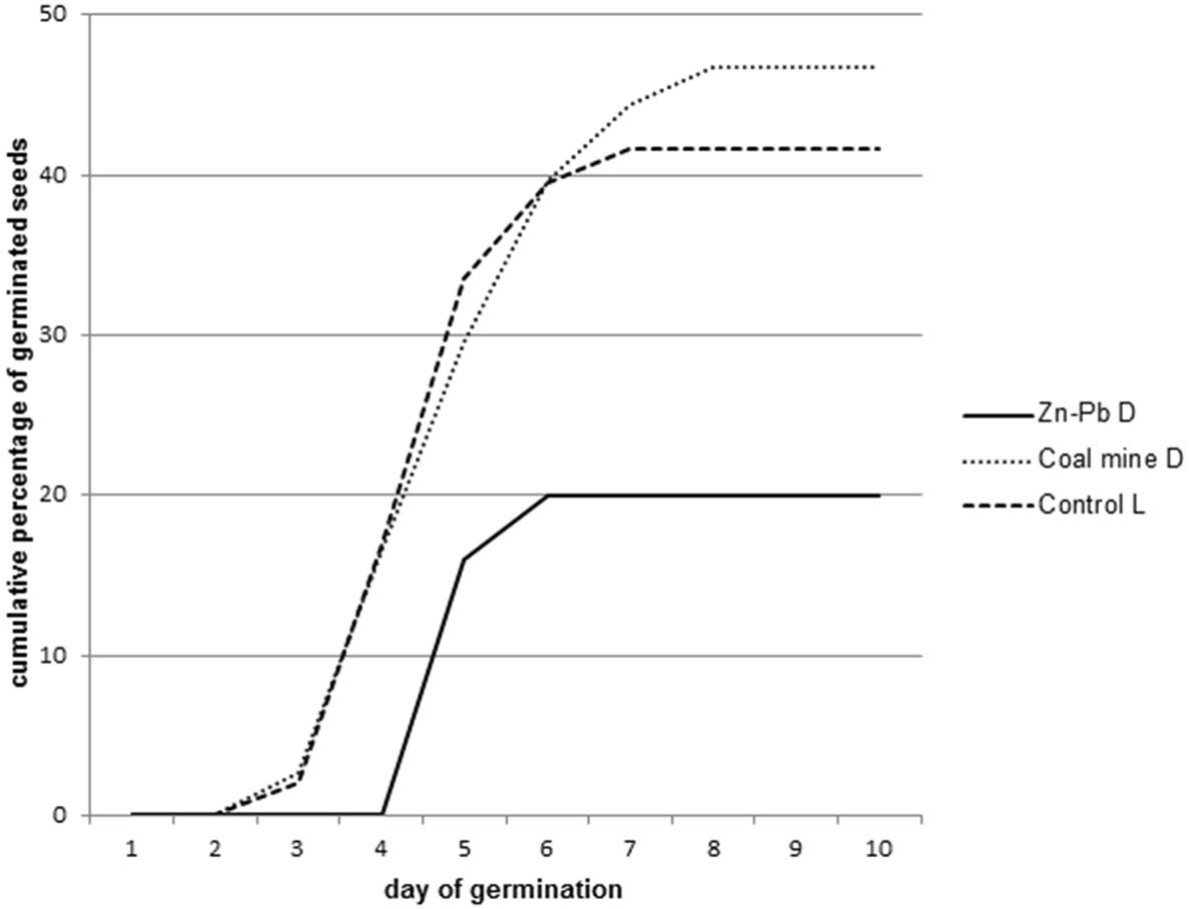
Cumulative percentage of germinated seeds during the 14 days of the experiment on the polluted Zn-Pb D, Coal mine D and the control site (Control L).

Different patterns of the germination of *B. pendula* seeds were found at the examined post-industrial and the Control L sites. Almost all the seeds from the Zn–Pb D germinated on the 5th day of the experiment. By contrast, seeds from the Coal mine D and the Control L site started to germinate on the 3rd day. They germinated gradually within 5 days, similarly to seeds from the unpolluted sites (Control L) (Fig. 3).

The vigour of a single *B. pendula* seed significantly differs with respect to the polluted (Zn–Pb D; Coal mine D) and the unpolluted sites (Control L). The highest MGT value was recorded in the case of seeds collected from the zinc-lead sites. By contrast, seeds from the coal mine spoil heap germinated 0.32 days faster and seeds from the control site 0.21 days faster than the Zn–Pb D ones (Fig. 3). The analysis showed that differences in the germination percentage index (G) between the research sites were statistically significant, and the highest value was recorded for the Control L site and the lowest for the Zn–Pb site (Table 3).

### Seedlings’ biometric traits

**Figure 4:**
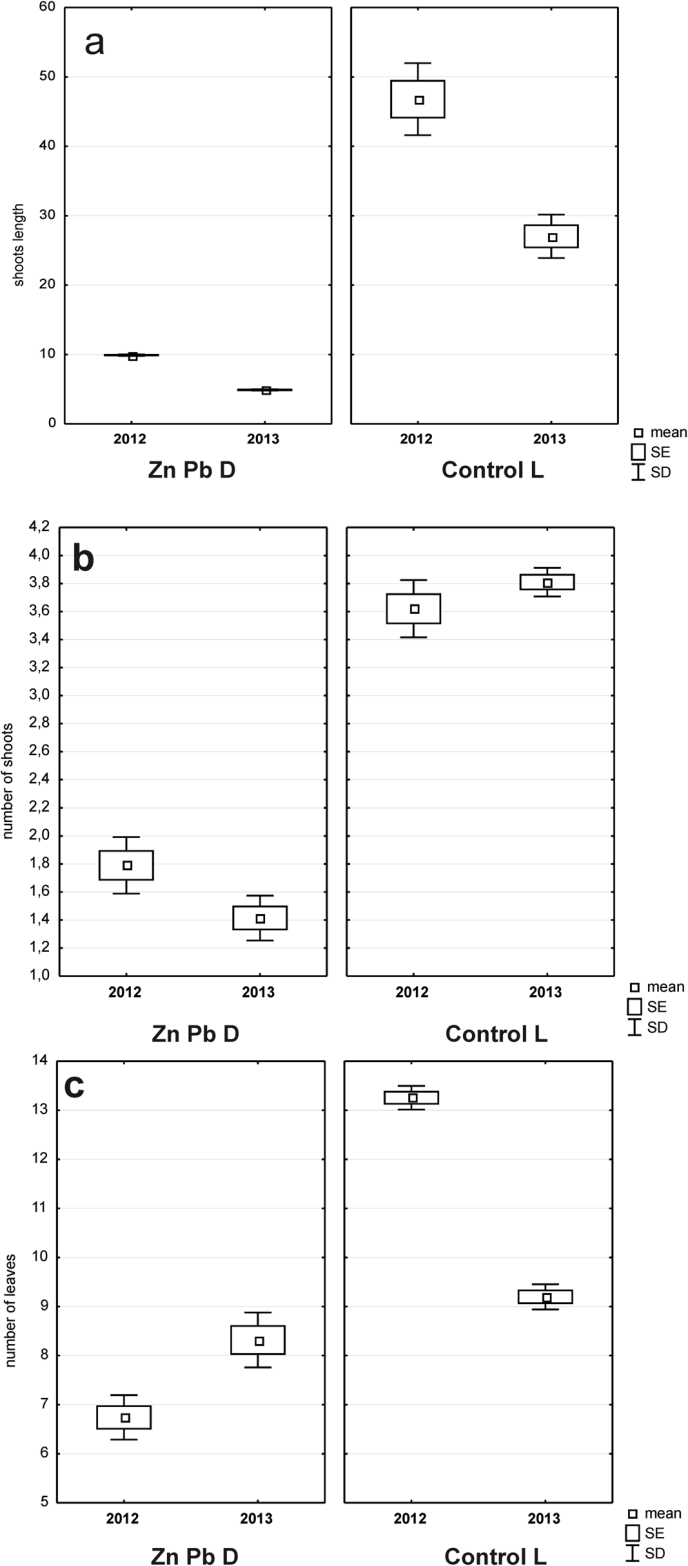
Comparison of the length of the birch seedling shoots (**a**), the number of birch seedling shoots (**b**), the number of birch seedling leaves (**c**) from the Zn-Pb D and Control L sites during two vegetative seasons.

The biometric studies of the above-ground shoots revealed statistically significant differences between seedlings from the Zn–Pb D in comparison to the Control L in terms of shoot length, shoot number and leaf number (Fig. 4). We did not observe any birch seedlings on plots established on the Coal mine D. The shoot length was two times higher on the control plots (Control L) than on the Zn–Pb D plots (Fig. 4a). The numbers of shoots and leaves were two times higher in the case of Control L. Seedlings from the unpolluted sites had 6 more leaves on average than seedlings from the zinc-lead heap (Zn-Pb D) (Fig. 4b,c). The results of Generalized Mixed Models show that the Zn-Pb or the unpolluted Control L sites have stronger effects on all the examined biometric traits of *B. pendula* seedlings (Table 4) than vegetative seasons (2012–2013). The largest differences between the seasons were found with reference to leaf number and shoot length. The number of leaves was lower on Control L in 2012 but increased in 2013, whereas on Zn–Pb D it was higher in 2012 but decreased in 2013. At both research sites the shoot length of seedlings was higher in 2012 but decreased in 2013. Only minor differences were found in the number of shoots between Zn–Pb D and Control L in vegetative seasons.

**Table 4:** The results of generalized mixed model (restricted maximum likelihood method) of *B. pendula* seedlings biometric traits. The research sites (Zn–Pb D, Control L) was treated as a fixed variable while vegetative seasons (2012, 2013) as a random variable.

Parameter	Fixed effects				Parameter	Random effects			
	Estimate	Std. Err	t-value	p-value		(Std. dev.)	AIC	BIC	logLik
(Intercept)	0.474	0.039	12.146	0.000	(Intercept)	0.034	224.754	202.988	-118.377
Shoot length	−0.413	0.055	−7.492		Season	0.171			
					Residual	0.153			
(Intercept)	0.232	0.037	6.182	0.000	(Intercept)	0.033	244.952	223.186	-128.476
Number of leaves	0.588	0.053	11.085		Season	0.264			
					Residual	0.147			
(Intercept)	0.352	0.038	9.229	0.000	(Intercept)	0.034	235.772	214.007	−123.886
Number of shoots	0.384	0.054	7.119		Season	0.054			
					Residual	0.151			

## Discussion

### Reproductive traits of birch

The biomass of a specimen determines its reproductive potential and thus when and how often it reproduces in a life cycle ^50,51^. This statement was indirectly confirmed by our research. The birch trees growing in the Katowice Forest Park (Control L) developed the largest number of leaves (412 leaves per branch) compared to the other research sites (Zn-Pb D; Coal mine D) (Table 2, Fig. 1). Moreover, the birch trees from the Control L sites also had the largest number of pistillate inflorescences as well as the longest staminate inflorescences and mature female catkin length. In pioneer plants, two mechanisms that control the allocation of energy resources of a specimen can be observed: for the growth and development and for the reproduction stage. This phenomenon is accompanied by a considerable reduction in growth intensity during the period of full flowering and fruiting ^52^, which was confirmed by our results of the PCA (Fig. 1). The biometric traits connected with pistillate inflorescences, empty nuts and the number of leaves significantly differentiated the birch trees of the polluted (Zn–Pb D, Coal mine D) and the unpolluted (Control L) sites. This could prove that individuals allocate a larger part of their energy expenditures to generative reproduction than to the development of vegetative parts only at sites with lower concentrations of heavy metals. The specimens of birch growing on the examined habitats did not develop a universal reproductive strategy. Despite the reduced number of all the flowers, the percentage of pistillate flowers was similar in the post-industrial areas (38% and 40% for Zn–Pb D and coal mine D, respectively). For Control L, 53% were pistillate flowers. The high level of soil toxicity detected in heavily polluted microhabitats frequently hinders generative reproduction, as in the case of the Zn–Pb heap site ^53^. In the light of the benefit–cost model, in such cases, when the value of both vegetative and generative reproduction in a given plant population is low, the increased allocation of resources to generative reproduction is higher. This permits seeds to be dispersed better in good habitat conditions ^54^. Consequently, plants in a polluted environment can be expected to have lower reproductive investment than plants growing in favourable conditions (a lower concentration of heavy metals, a higher level of N, P, K). Since the impact of severe pollution diminishes the soil nutritional quality due to the displacement of base cations of heavy metals, it is likely that the cost of reproduction will increase at polluted sites, thus decreasing the overall fitness of plants’ reproduction ^55^. Moreover, in the severely polluted habitats, the concentrations of N, P and K were lower compared to the unpolluted sites. We have confirmed this in our research because all of the values that are connected with the biometrical traits of *B. pendula*, as well as germination capacity, were found significantly lower for the heavy metal polluted sites compared to individuals that were growing at the coal mine spoil site and the unpolluted site.

The analysis of the individual components of the final reproductive effect indicated their immense flexibility, whereas the reactions of the plants to unfavourable factors, which led to low reproductive efficiency, are not always comprehensible. It is difficult, however, to consider the reproductive behaviour of the individuals or specific pioneer plants of any species such as birch separately from the general life strategy. Grime's concept of life strategies does not involve species that have become adapted to strong stress and intensive disturbances at the same time (see RDA results, Fig. 2). However, greater capabilities for preserving an occupied territory through a shallow but extensive root system and capabilities for tolerating a nutritional deficiency, low moisture content and high temperatures can facilitate the growth of birch populations in stress-inducing habitats that are vulnerable to disturbances ^56^.

### Pollen and seed viability

The influence of stress factors (e.g. acid precipitation, UV radiation, CO, O_3_, and SO_2_) on the quality of pollen was confirmed by microscopic observations of the development of birch flowers ^57,58^. Elevated CO_2_ and O_3_ can dramatically affect flowering, seed production, and seed quality of paper birch, affecting reproductive fitness of this species^14^. Our research revealed that the germination capacity of *Betula* pollen was significantly (70–80%) reduced at the Zn-Pb D site and 20% at the Coal mine D site compared to the Control L site (Table 3). The two SEM images (ESM_1 and 2) show a high and a low number of pollen grains on the flower style. A germinating pollen grain and its pollen tube release certain highly physiologically active substances of into a pistil, which trigger the additional physiological polarisation of the generative organs and the acceleration of the embryo sac's development ^59^. Thus, both the quality and the amount of pollen influence the setting of seeds. In our results, the smallest number of staminate inflorescences and the largest number of empty birch nuts were recorded in the trees growing on the Zn-Pb D in contrast to the Coal mine D (Fig. 1, 2, Table 2). In stressful conditions, flowers are pollinated with their own pollen and then parthenocarpic seeds develop ^60^. Although the extent of developmental depression related to inbreeding is diverse, seeds capable of further development are usually created.

Apart from weather conditions, the fruiting of birch trees is influenced by the availability of the energy resources that are accumulated by a tree. Changes in the climatic and edaphic conditions induce modifications in the chemical composition and the physiological characteristics of seeds, whereas in disturbed habitats where conditions can change in an unpredictable manner, different maturation time in seeds, even those that come from the same fruit, is quite a common phenomenon ^11,61^. Consequently, seeds do not germinate simultaneously and this increases the chance that successful progeny will develop. The time of seed dispersal, particularly the time of seed germination, is no less important than the place at which a young organism starts. The polymorphism of birch seeds, in terms of the duration of the dormancy period, which depends on habitat conditions, is reflected in the sowing quality of seeds and the dynamics of their germination. In our research, the seeds of the birch trees from the Zn-Pb D were characterised by the lowest germination capacity (10.40% to 21.60%), as well as by the highest mean germination time (Table 3, Fig. 3). The decreasing germination rate of seeds and the reduced percentage of germinated seeds in a sample are obvious physiological symptoms of the decreasing viability of seeds. An immature seed embryo or the influence of inhibitors might be the underlying cause of the reduced sowing value of seeds. This can be evidenced by the extension of the phase of maximum germination of seeds from four to six days for the diaspores on the zinc slag heap (Zn–Pb D; Fig. 3). The low quality of seeds results from the influence of extreme habitat conditions on the parental organisms and on the course of the reproductive phase. Moreover, the pattern of seed germination was different with respect to the origin of the seeds. All of the seeds germinated on the fifth day in the case of Zn–Pb D, and where only fully developed seeds were taken into account, the percentage of germinated seeds was the highest. This may be evidence of the excellent suitability of this species for colonising and settling post-industrial areas.

### Biometric traits of birch seedlings

Unfavourable environmental conditions, e.g. soil contamination, low pH or low nutrient status, can result in poor development of birch seedlings ^62^. As in the aforementioned results, we observed that after seed shading seedlings already appeared in the Control L in the same year. By contrast, at the lead and zinc heap sites (Zn–Pb D), seedlings appeared in the next spring, mainly in gutters where the moisture was higher. The Generalized Mixed Model confirms that stress caused by habitat conditions has a greater impact on selected biometric features of *B. pendula* seedlings than the particular growing season in which the measurements were taken (Table 4). We did not find birch seedlings on the coal mine spoil site (Coal mine D). This is probably connected with the fact that birch seeds and young seedlings are extremely sensitive to soil moisture and the uppermost layer started to dry off during summer months.

Moreover, the content of heavy metals was considerably high at the Zn–Pb site (Table. 2), which influenced the worse biometric parameters of seedlings. A significant characteristic of many plant populations that are tolerant to metals is their weaker growth compared to plants from uncontaminated soils, which is often accounted for by energy costs or the mechanisms of tolerance to metals ^63^. Similarly, *Betula pubescens* subsp. *czerepanovii* birch seedlings growing near a copper-nickel smelter were smaller and produced smaller leaves when grown in clean soils ^35^. In contrast to the reference site, the birch seedlings growing at the heavy metal contaminated sites were characterised by a smaller number of branches and grew only to one third of the control height. The reduction in the leaf number in a polluted area is connected with the plants’ slow, early-arrested growth, which may be caused by a generally low vitality of trees that results in a smaller surface for assimilation. In the Katowice Forest Park (Control L) the number of birch seedlings shoots was three times higher compared to the post-industrial areas, which may be explained as being the response of the birch to the high population density at unpolluted sites (Fig. 4). The analyses revealed that the Zn–Pb site (Zn–Pb D) differed significantly from the other sites regarding the height of seedlings. As a consequence of the high density, seedlings have to reach the greatest possible height in order to shade other specimens and win the competition for light.

Several research deal with biotic factors that have influence on the interactions between *Betula pendula* and other pests. They revealed that percentage leaf removed by the chewing insects was significantly higher on the ant-free trees than on the foraged trees. In result unforaged trees had significantly fewer leaves per shoot than had foraged trees ^64^. Although both abundance and species richness of leaf miners difered among birch genotypes at the tree level, birch genetic diversity had no significant effect on miner abundance and species richness at the plot level. Instead, birch genetic diversity affected leaf-miner β-diversity with species turnover being higher among trees within genotypic mixtures than among trees within monoclonal plots ^65^. Some pests can affect birch e.g. European hornet (*Vescpa crabro germana)* may cause forks ^66,67^. *Phytobia betulae* causes black irregular-shaped configuration within the core wood. Other insect such as moths, aphids or insect larvae (caterpillars) can cause leaf damage however it has not significant impact on tree health or growth ^68^.

## Conclusions

A thorough knowledge of the reproductive biology of the *Betula pendula* Roth population and its response to variable environmental conditions can provide a practical basis for introducing this species into areas that have been affected by long-lasting industrial activity (excavation and processing of hard coal, lead and zinc ores). Owing to increased genetic flexibility and under the influence of environmental selection pressure, *B. pendula* is able to develop traits that facilitate its survival. The use of birch seeds from heavily polluted areas can possibly increase the success of land reclamation, the aim of which is to create woodland habitats and protect such sites from water and wind erosion, as well as to prevent the leakage of heavy metals into the soils and ground waters. In order to survive, it can allocate less energy for growth, with more for reproduction, and use different patterns of pollen and seed germination. Such adaptations can occur within a relatively short period of time, thereby providing genes of an adaptive nature that are present in the founding population. Some reclamation treatments should probably be undertaken in order to improve the substrate quality as well as water availability (by covering it with a layer of garden soil) ^69^. The results of long-term studies on the settlement processes of specific species on post-industrial heap sites may facilitate planning activities that are related to the successful restoration of such degraded areas.

## Supplementary Information

Supplementary Figures.
