# Supplementary material for: Reproduction strategies of the silver birch (Betula pendula Roth) at post-industrial sites
Source: Sci Rep. 2021 Jun 7;11:11969. doi: 10.1038/s41598-021-91383-0 (PMC8185007; doi:10.1038/s41598-021-91383-0)

Fig. S1

Does generative reproduction enhance the success of *Betula pendula* Roth at post‑industrial sites? Izabella Franiel and Agnieszka Kompała-Bąba, University of Silesia in Katowice,

e-mail: [izabella.franiel@us.edu.pl](mailto:izabella.franiel@us.edu.pl)

A fragment of *B. pendula* Roth style originated from Zn-Pb D female catkin. It is visible a lack of pollen grains in SEM


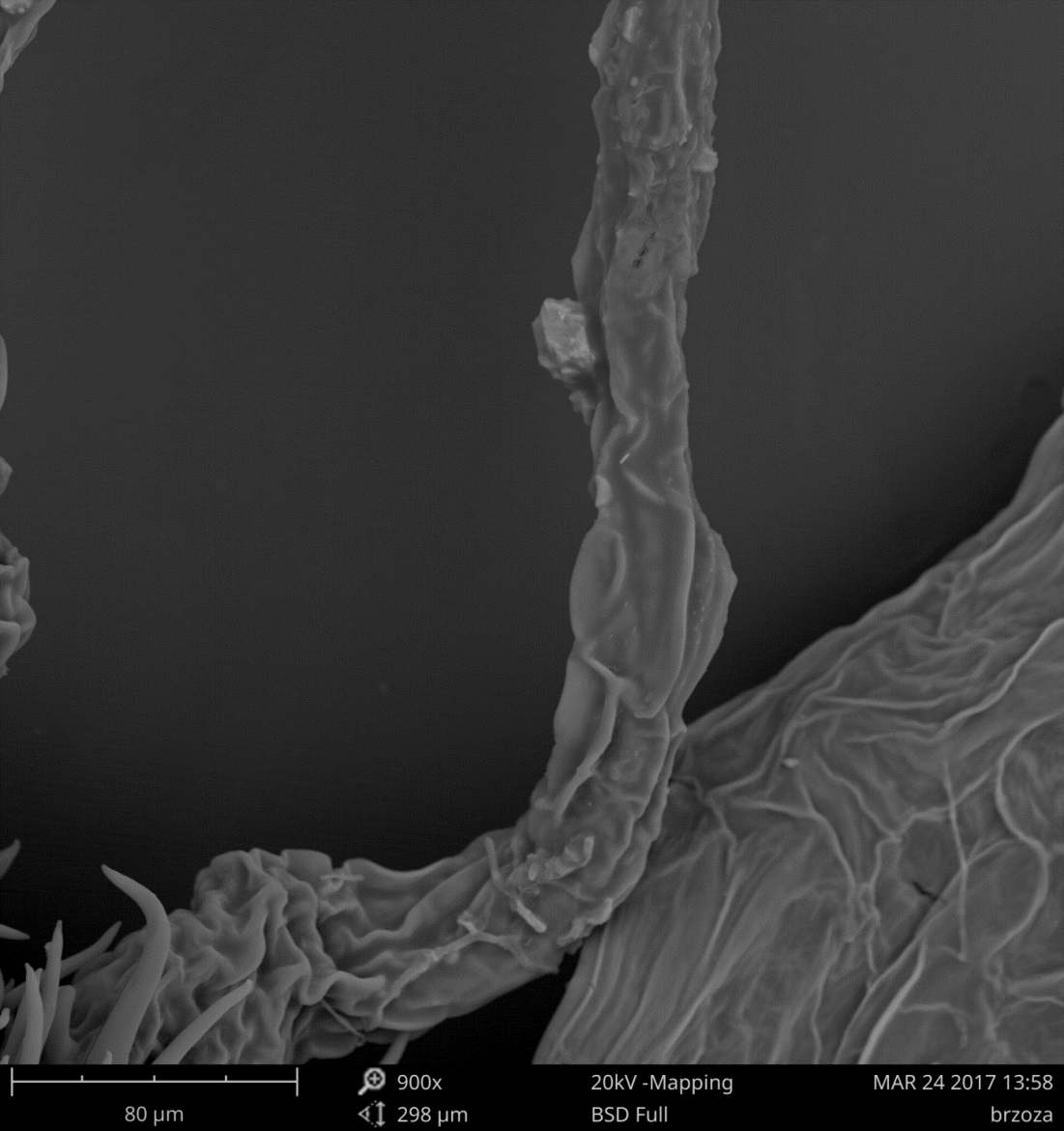


Fig. S2

Does generative reproduction enhance the success of *Betula pendula* Roth at post‑industrial sites? Izabella Franiel and Agnieszka Kompała-Bąba, University of Silesia in Katowice,

e-mail: [izabella.franiel@us.edu.pl](mailto:izabella.franiel@us.edu.pl)

A fragment of *B. pendula* Roth style that originated from the Katowice Forest Park research plot (Control L) female catkin. A large number of pollen grains is visible on a style in SEM


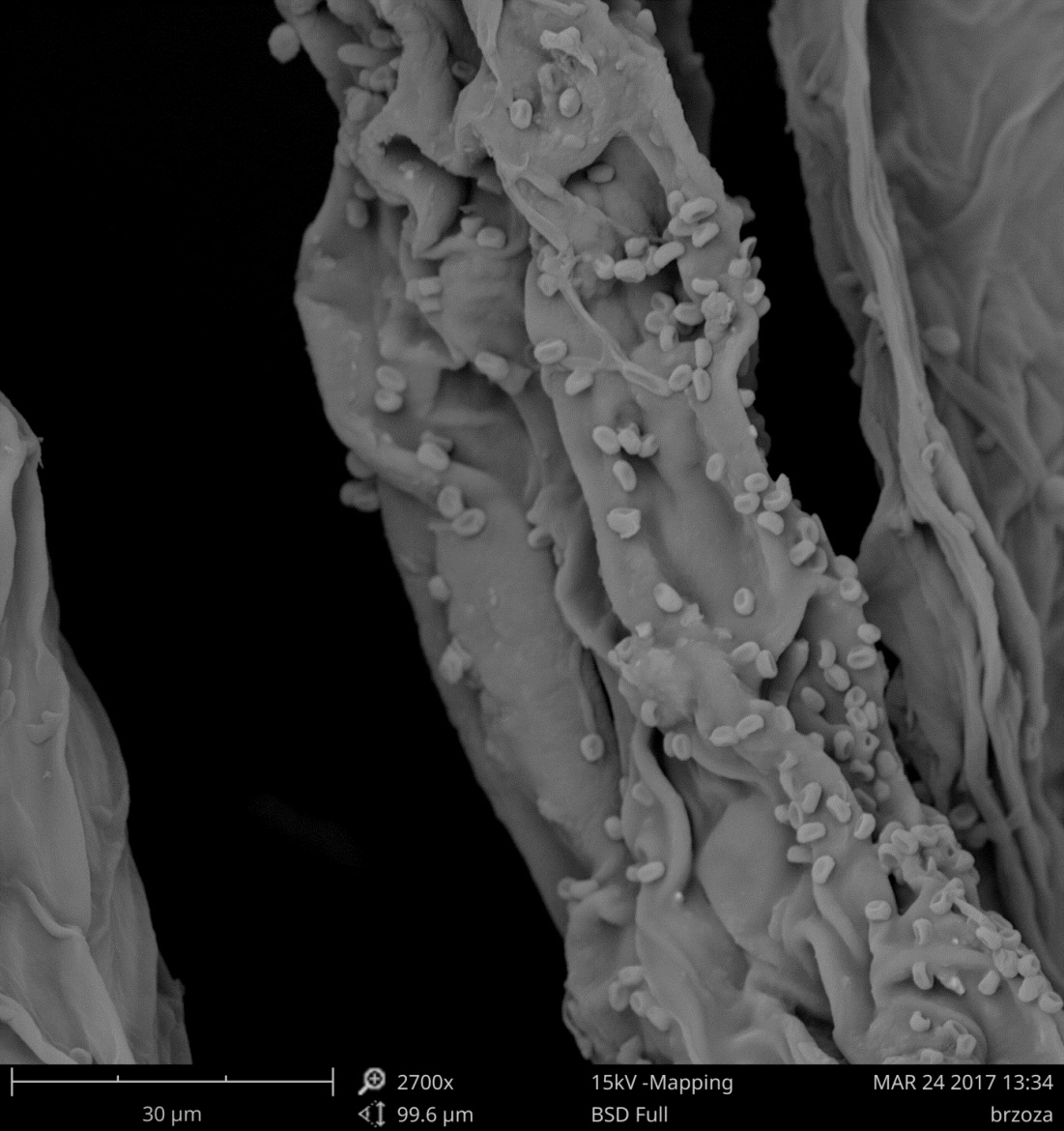

Supplement: Supplementary file 1 — Supplementary Figures. [file 41598_2021_91383_MOESM1_ESM.docx]
